# Institutional partnerships: results from a scoping review of promising strategies to promote diversity within the academic health sciences

**DOI:** 10.1186/s12909-025-08096-5

**Published:** 2025-10-31

**Authors:** Christian Herrera, Krystal Abbott, Anthony Trinidad, Michelle Cheng, Sheri Markle, Margarita Alegria, Idia B. Thurston

**Affiliations:** 1 Department of Psychological Sciences, University of Missouri, Columbia, Columbia, MO USA; 2 CHANGE Lab, Institute for Health Equity and Social Justice Research, Northeastern University, 322 INV, Boston, MA 02186 USA; 3 Heller School for Social Policy and Management, Brandeis University, Waltham, MA USA; 4 Disparities Research Unit, Department of Medicine, Mongan Institute, Massachusetts General Hospital, Boston, MA USA; 5 Department of Medicine and Psychiatry, Harvard Medical School, Boston, MA USA; 6 Departments of Public Health & Health Sciences and Applied Psychology, Northeastern University, Boston, MA USA

**Keywords:** Institutional partnerships, Diversity, Recruitment, Retention, Academic health sciences, Implementation

## Abstract

**Background:**

Within the academic health sciences, scholars from racially and ethnically minoritized backgrounds continue to remain underrepresented. Establishing institutional partnerships is one strategy that has accumulated evidence as a mechanism to diversify the academic health sciences and professionals within the health care system. Despite the evidence supporting institutional partnerships, guidance on implementing these partnerships is lacking within existing literature. The present study synthesizes existing literature and provides best practices for implementing institutional partnerships.

**Methods:**

A pre-registered scoping review examined evidence-based strategies to advance racial equity, reduce institutional racism, and increase the recruitment, retention, and promotion of faculty, staff, and students of color. Articles that met the inclusion criteria were screened, and cross-cutting elements for establishing institutional partnerships were extracted.

**Results:**

Of a total of 5578 articles that were screened and 366 that were reviewed, 6 articles were relevant to establishing institutional partnerships. Institutional partnerships that successfully diversified the academic health sciences were grouped into 3 categories: institutional partnerships between historically white institutions and minority serving institutions, institutional partnerships to create career advancement opportunities, and institutional partnerships as a form of resource sharing. Best practices for establishing institutional partnerships were identified.

**Conclusions:**

These best practices offer a hopeful path for ensuring a diverse workforce in health care. Methodological weaknesses of existing literature were highlighted, providing guidelines and direction for future research.

Since the summer of 2021, there has been an uptick in the number of institutions and accreditation bodies in the academic health sciences issuing calls to action, all outlining the immediate imperative to diversify the body of students enrolled in existing programs. In 2015, the Liaison Committee for Medical Education (LCME), one of the major organizations responsible for medical school accreditation, established standard 3.3 which outlined components of a diverse medical school learning environment including a mission-appropriate diversity policy, systematic recruitment and retention activities to achieve diversity outcomes, methods to evaluate these activities, and demonstrated effectiveness of these activities in achieving diversity outcomes [1]. Similarly, the Accreditation Council for Graduate Medical Education (ACGME) implemented common program requirement I.C., which detailed the importance of mission-driven systematic recruitment and retention of diverse residents, faculty, and staff [2]. Although both standards were suspended in 2025, in part to avoid conflict with the current legislative environment, they institutionalized the necessity of cultivating a diverse workforce among medical students and faculty [3, 4]. The American Association of Colleges of Nursing also released a statement reaffirming its commitment to recruiting and retaining a diverse workforce [5]. Action was taken by the American Public Health Association (APHA) by not only voicing their commitment to diversity, equity, and inclusion in written statements but also launching a joint initiative with Kaiser Permanente to diversify student pathways to public health careers and ultimately the composition of public health leadership [6].

These calls to action parallel the rapidly changing demographic composition of the U.S., which is predicted to further diversify in the coming years. By 2060, approximately 59.5% of the people in the U.S. are expected to identify with the global majority^1^ [8]. Between 2016–2060, all global majority populations will experience substantial growth including individuals identifying as Black/African American (33.5%), American Indian and Alaska Native [AIAN] (43.6%), Asian (171.1%), Native Hawaiian and Other Pacific Islander [NHPI] (50.3%), Multiracial (215%), and Hispanic groups (122%) [8]. These calls to action to diversify the academic health sciences are further backed by existing research demonstrating the benefits of diversity in the context of student learning outcomes and lifesaving clinical outcomes.

## Benefits of diversity

Racial and ethnic diversity has been linked to improved student learning outcomes and real-world clinical applications for racial and ethnic minorities, highlighting the necessity of recruiting and retaining a diverse student workforce.

### Learning and education outcomes

Gurin et al. [9] examined the association between informal interactional diversity (i.e., the quality and quantity of interactions with racially and ethnically diverse populations outside of the classroom) and classroom diversity (i.e., curriculum incorporating activities geared towards understanding different racial/ethnic groups) to learning outcomes among college students at a national level. Exposure to informal interactional diversity, which included attending cultural awareness workshops, discussing racial issues, and socializing with a person of a different race, was significantly associated with intellectual engagement and academic skills for white, African American, and Asian American students, and significantly related to academic skills among Latine/a/os. Classroom diversity, which included “enrollment in an ethnic studies course”, was positively related to intellectual engagement and academic skills among white and Latine/a/o students; however, it was negatively associated with academic skills among African American students and not significantly related to learning outcomes among Asian American students.

Diversity has been shown to benefit white students and Underrepresented and Minoritized (URM) students. White medical students at schools with greater racial and ethnic diversity had statistically greater odds of higher self-rated cultural competence and promoting access to equitable care than those with fewer URMs [10]. Among medical students, exposure to racial and ethnic diversity was positively correlated with agreeing with the statements: “My knowledge or opinion was influenced or changed by becoming more aware of the perspectives of individuals from different backgrounds”, and “the diversity within my medical school class enhanced my training and skills to work with individuals from different backgrounds” [11]. It should also be noted that this association was strongest for URM students [11]. Furthermore, racial diversity among team-based learning cohorts was positively and significantly associated with group readiness assurance test (GRAT) scores and the perception that diversity contributed to GRAT performance [12]. Among fourth-year dental students, exposure to a diverse student body was significantly associated with student confidence in working with diverse patients [13].

### Clinical and real-world applications of diversity

Cultivating diversity in the classroom prepares all health care professionals (irrespective of race and ethnicity) to work with and treat diverse populations [9–13]. Beyond providing educational benefits, a diverse workforce is essential in serving and improving outcomes for minoritized individuals who systematically experience a disproportionate health burden. Indeed, health care professionals from minoritized racial and ethnic groups are more likely to work with minoritized communities [14–19], deliver lifesaving care and reduce mortality rates through cultural concordance [20–25], and provide more efficient patient care and hospital operations [26].

URM medical students are more likely to express interest in working with underserved populations upon entering and graduating medical school relative to non-URM individuals [14]. URM dentists and medical doctors have demonstrated a higher likelihood of serving racial and ethnic minoritized patients compared to their non-URM peers. Indeed, dentists from URM backgrounds reported serving more patients of their own racial or ethnic group relative to other racial or ethnic groups [15] as well as less-educated and lower-income patients [16]. Mertz et al. [15] found that, on average, Black dentists had a patient population that was 44.9% Black, AIAN dentists had a patient population that was 20.4% AIAN, and Hispanic and Latine/a/o dentists had a patient population that was 41.8% Hispanic/Latine/a/o. This pattern of serving minoritized populations persists with URM medical school graduates and physicians, who are more inclined to serve minoritized and disadvantaged populations than non-URM health professionals [17]. A systematic review found that, relative to non-URMs, physicians identifying as URM were more likely to work in underserved areas and serve a greater number of patients with minoritized identities [18]. The percentage of underserved patients Black, Hispanic, and Asian physicians saw was 60.1%, 62.7%, and 47.9%, respectively, while white physicians had an underserved patient pool of 24.8% [19].

There are stronger associations between racially concordant professional-patient dyads and increased utilization of needed health services, an increase in attended doctors' visits, greater satisfaction compared to racially discordant dyads, and reduced mortality rates for Black newborns. LaViest et al. [20] found that white and African American patients with racially concordant physicians were significantly less likely to avoid needed health services and more likely to attend doctor visits relative to race-discordant professional-patient dyads. Asian patients with concordant providers were statistically more likely to seek preventative care, care for new problems, and care for ongoing problems relative to discordant dyads [21]. A similar study examined the effects of patient satisfaction in race-concordant doctor-patient dyads [22]. African Americans, Hispanics, and Asian American patients who had racially concordant providers reported higher levels of satisfaction compared to those who had racially discordant providers. There is consistency in the literature about race concordance and favorable professional-patient communication satisfaction [23]. An increase in Black primary care physicians was associated with an increased life expectancy and reduced mortality rate for Black individuals [24]. Furthermore, racial concordance has been linked to reduced mortality rates for Black newborns [25]. A study by Greenwood et al. [25] found that the mortality rate for Black newborns is reduced by 39%—58% when cared for by a Black physician, relative to a white physician. Race concordance in patient/provider dyads is essential to expanding access to health services and improving health outcomes.

Workforce diversity within the hospital setting is also associated with higher levels of hospital efficiency. In a study by Lee et al. [26], they found that hospitals exhibiting higher levels of racial diversity had significantly higher efficiency than hospitals with lower levels. States within the U.S. with the highest proportions of racially and ethnically minoritized registered nurses (e.g., Hawaii, with a rate of 68.2% compared to Maine, with a rate of 3.3%) have shown a decreased risk of severe adverse maternal outcomes ranging from 20% for Black mothers and 50% for Asian and Pacific Islander mothers [27], further exemplifying the necessity for a diverse hospital workforce to attain a healthier population.

The exact mechanism through which diversity and racial concordance are linked to positive outcomes is not fully understood. However, one possible explanation is found in the concept of structural competency. As defined by Mertz [28], structural competency is the “trained ability to discern how a host of issues defined clinically as symptoms, attitudes, or diseases…also represent the downstream implications of a number of upstream decisions about such matters as health care and food delivery systems, zoning laws, urban and rural infrastructures, medicalization, or even about the very definitions of illness and health”. It may be that a diverse workforce is more readily able to strive for components of structural competency, as diverse physicians and providers may come from similar backgrounds and lived experiences of the individuals that they serve. Diverse health care professionals may be best positioned to understand the systemic drivers of health disparities and thus positioned to provide the highest quality of care and service for all, and particularly for those who are minoritized.

## Lack of diversity in the academic health sciences

Despite the calls to action to recruit and retain a diverse health care workforce and research demonstrating the benefits of diversity, the racial and ethnic diversity of graduate programs in public health, nursing, dentistry, and medicine remains low. The lack of diversity is a result of historical and ongoing acts of racism that have attempted to systematically exclude minoritized individuals from pursuing careers in the academic health sciences [29]. Redlining [30], reforms brought on from the Flexner Report [31], exclusion from professional societies [32], “academic redlining” [33], and the experience of microaggressions [34–36] continue to prevent minoritized scholars from entering the academic health sciences.

Graduate student enrollment in public health programs between 1996 and 2016 demonstrated marginal increases among minoritized scholars [37]. In those 20 years, the number of enrolled students in public health programs who identified as Asian increased by 5%, 2.7% for Black students, and 3.5% for Hispanic students, while the percentage of Native American and white students decreased by 0.3% and 10.9%, respectively [37]. It should be noted that despite the 10.9% decrease in white student enrollment, white students represented the largest percentage in both 1996 (71%) and 2016 (60.1%). Doctoral graduation rates between 1996 and 2016 mirrored the marginal increases seen in enrollment as the number of Ph.D. graduates who identified as Asian increased by 5.6%, 3.9% for Black students, 4% for Hispanic students, and a decrease of 0.3% for Native American students [37].

Diversification of the nursing workforce has consistently lagged behind the racial diversity of the U.S population [38–40]. Hynson et al. [41] analyzed data from two surveys documenting changes in diversity between 2008 and 2018 (National Sample Survey of Registered Nurses [NSSRN]) and 2010 and 2019 (American Community Survey [ACS]). Both surveys reported statistically significant growth in the number of Black/African American and Latine/a/o RNs and nurses with an MS/PhD. The NSSRN also reported significant growth in the number of NH/PI RNs. The percentage of AIAN RNs did not significantly increase. Furthermore, AIAN and NH/PI nurses with an MS/PhD did not significantly increase [41].

Among dentistry students, the number of first year URM students paints a similar picture to that of public health and nursing. In 2019, URM students made up only 16% of the enrolled student population, including Hispanic/Latine/a/o (10%), Black/African American (5.8%), AIAN (0.1%), and NHPI (0.1%) [42]. The percentage of URM graduates was also relatively low among Hispanic/Latine/a/o (8.4%), Black/African American (4.9%), AIAN (0.4%), and NHPI persons (0.2%). Majerczyk et al. [43] found that across applicants, matriculants, and degree conferrals for the Doctor of Dental Surgery (DDS) and Doctor of Dental Medicine (DMD), the number of URMs increased from 2003–2019; however, the percentage of AIAN, Black/African American, Hispanic or Latine/a/o, and NHPI groups remained lower than the overall U.S. population.

According to the Association of American Medical Colleges [AAMC] [44], matriculants into medical school during 2019 were predominantly white (49.9%), followed by Asian (22.1%), Multiracial (9.5%), Black/African American (7.1%), Hispanic/Latine/a/o (6.2%), AIAN (0.2%) and NHPI students (0.1%). Graduation rates from 2019 reflect similar statistics to matriculation; however, all groups except white students reported lower graduation rates, including Asian (21.6%), Multiracial (8%), Black/African American (6.2%), Latine/a/o (5.3%), and AIAN (0.2%), suggesting specific barriers to retention for these groups [44].

The problem of successfully recruiting and retaining diverse scholars in the academic health sciences remains. Several promising strategies have been identified in the literature to increase diversity in the academic health sciences, including policy changes, holistic admissions, embedding anti-racism, pathway programs, and more [45]. Institutional partnerships have been identified as a promising strategy to increase diversity within the academic health sciences [45] and have received empirical support within the literature [46, 47]. Furthermore, theoretical frameworks aimed at increasing the recruitment and retention of URMs in the academic health sciences posit institutional partnerships as the cornerstone strategy, or the foundation upon which other strategies (e.g., pathway programs, holistic admissions, emotional and social support) are built [48]. This paper examines institutional partnerships as one promising strategy to close matriculation and graduation gaps.

## Institutional partnerships as a mechanism to diversify the field

Institutional partnerships between historically white colleges/universities (HWCU) and minority-serving institutions (MSI) may function as a necessary, corrective response to systemic inequities that have prevented minoritized scholars from entering the academic health sciences. The 1910 Flexner Report [31], which prompted the closure of all but two historically Black medical schools in the United States, exemplifies the long-term impacts of educational exclusion, with an estimated loss of up to 35,315 potential Black physicians by 2019 [49]. Structural racism has also contributed to the persistent underfunding of Historically Black College/Universities (HBCUs) through discriminatory allocation practices such as the states' failure to provide matching funds under the 1890 Second Morrill Act and exclusion from receiving federal funding, resulting in $12.8 billion in underfunding for HBCUs over the past three decades alone [50]. By leveraging shared resources, institutional partnerships could mitigate systemic barriers and promote a pathway to structural change, thereby serving as a sustainable mechanism for diversifying the field.

The “Framework to Successfully Recruit and Retain URMs in Health Professions” was developed by Toretsky et al. [48]. This framework is applicable across multiple disciplines and consists of three core components: 1) forming institutional partnerships, 2) tailoring student support and academic success, and 3) engaging faculty/institutional change [48]. Forming institutional partnerships is considered the “cornerstone” of this framework and serves as the foundation for providing student support and institutional change. This is consistent with past literature demonstrating the effectiveness of institutional partnerships in diversifying the academic health sciences [46, 47].

Goines et al. [46] reported on the success of a partnership between Morehouse School of Medicine (MSM), an HBCU, and Emory Emergency Medicine (Emory EM), first created in 1999. The partnership was developed to increase the number of underrepresented students matching into emergency medicine. Students from MSM, a school that did not have an emergency medicine specialization or department, were provided mentorship and support by faculty and staff from Emory EM, equitable rotation opportunities, and institutionalization of the partnership by offering it as a course elective [46]. There was a significant increase in the number of MSM students matching into EM after the program in 1999 (6.65%) compared to before the program (3.01%). The study did not report on the specific racial and ethnic composition of the students who matched into EM, but assumed the population was diverse, given the student population at MSM.

Another example of a successful institutional collaboration is the Undergraduate Medical Academy (UMA), which was between Texas A&M Health Science Center, a Historically white College/University (HWCU; [51]), and Prairie View A&M University (an HBCU) to increase the number of underrepresented scholars in medicine [47]. Students admitted to the UMA received various support, including academic counseling, MCAT prep, and tutoring; a faculty and medical student-led seminar series; site visits from medical students; and summer programs that provided laboratory experience and shadowing opportunities. The UMA involved leadership from both institutions and an external review board with an aligned mission of increasing diversity in medicine. The UMA had a program retention rate of 91.7% and a medical school acceptance rate of 64%.

Institutional partnerships may serve as a promising strategy to achieve racial equity in the academic health sciences. However, there are two significant barriers in existing research. First, there is no comprehensive understanding of how institutional partnerships have been used to diversify academic health sciences successfully. Second, there is no consensus on best practices for establishing institutional partnerships. As described in the “Framework to Successfully Recruit and Retain URMs in Health Professions” [48], there is a lack of specific action steps that institutions seeking to establish partnerships may utilize. Thus, the present study aims to accomplish two goals to address these literature gaps. First, we provide an overview of existing research that has utilized institutional partnerships to increase racial and ethnic diversity within the academic health sciences. These studies were identified from a larger scoping review conducted to address the following questions: (i) What strategies have been implemented to advance racial equity in the academic health sciences? (ii) What strategies have reduced institutional racism in the academic health sciences? (iii) What strategies have increased the recruitment, retention, research productivity, and promotion of underrepresented students, faculty, and staff in the academic health sciences? Second, we offer best practices for building institutional partnerships to guide future implementation efforts.

## Methods

### Protocol and registration

A scoping review protocol was preregistered [52] and conducted following guidance outlined by Peters et al. [53]. A PRISMA-ScR checklist was also utilized to ensure accurate reporting [54]. The protocol can be accessed through OSF at the following web address: 10.17605/OSF.IO/H3SXF.

### Eligibility criteria

Eligible articles involved studies from any country that: 1) had a population focus on underrepresented faculty, students, and staff of color, as well as institutional leaders of all backgrounds who are involved in decision-making processes, 2) evaluated strategies that address structural racism and advance racial equity in academic health sciences, 3) occurred within health science/pre-health programs affiliated with colleges or by entities with substantial influence on the field of health science (i.e., professional associations, private testing organizations), 4) were quantitative, qualitative, or mixed methods in nature, and 5) were written in English.

### Information sources

From August 28, 2021 – November 21, 2021, six databases were searched including PsycInfo, MEDLINE (PubMed), ERIC, Education Source, Academic Search Ultimate, and CINAHL (EBSCO) for studies conducted between 1964–2021. A supplemental search was conducted on September 9, 2024 to determine if any new literature regarding institutional partnerships had been published since the original scoping review.

### Search

Search terms were developed in relation to the key concepts of justice, diversity, equity, and inclusion, higher education, health sciences, intervention, racial/ethnic categories, and faculty, staff, students, and administrators. See Table 1 for the full search terms utilized in PsycInfo and replicated across the other databases. 

**Table 1:** List of terms used in the scoping review search strategy in Psych Info database

Search	Query (Title/Abstract)	Records retrieved
**#1** (JDE&I)	diversity OR diverse OR equity OR inclusion OR inclusiv* OR “inclusive leadership” OR multicultural* OR “organizational climate” OR “campus climate” OR “institutional climate” OR cultural OR multicultural* OR “ethnic diversity” OR “racial relations” OR “racial attitudes” OR “ethnic relations” OR “ethnic attitudes” OR ethnic* OR “cultural sensitivity” OR “racial disparit*” OR “racial privilege*” OR “racial bias*” OR racism OR antiracism OR antiracist OR “implicit bias” OR discrimination OR prejudice OR microaggression OR justice OR “procedural justice” OR “racial justice” OR “distributive justice” OR fairness OR marginalization OR sex discrimination” OR “gender discrimination” OR “age discrimination “ OR “employment discrimination” OR “disability discrimination” OR sexism OR “gender gap” OR “gender equality” OR ageism OR “affirmative action” OR “pay equity” OR marginalization OR exclusion OR minoritization OR “equal employment opportunity”	604,711
**#2** (Higher ED)	academic OR academia OR college* OR “higher education” OR college* OR campus* OR “graduate school*” OR “graduate training” OR “graduate education” OR “doctoral training” OR “doctoral education” OR “postgraduate training” OR “postgraduate education” OR “medical training” OR “medical education” OR “nursing training” OR “nursing education” OR “medical internship” OR “medical residency” OR (“historically black” OR tribal) N3 (college* OR universit*) OR (“minority-serving” OR “Hispanic-serving”) N3 (college* OR universit* OR institution*) OR “academic medical center*”	344,731
**#3** (Health Science)	“medical science*” OR “paramedical science*” OR “behavioral science*” OR pharmacy OR nursing OR dentistry OR psychology OR neuroscience* OR psychiatry OR health OR “public health” OR “allied health” OR “health science*”	965,356
**#4** (Intervention)	outreach OR “program* OR strateg* OR “best practice*” OR policy OR policies OR “organizational change” OR “organizational behavior” OR recruitment OR selection OR evaluation OR training OR promotion OR tenure OR engagement OR “occupational mobility” OR “career development” OR “employee development” OR “professional development” OR mentor* OR “admission criteria” OR “college admission*” OR “educational measurement” OR approach OR initiative OR structural OR “structural level” OR Institutional level” OR internship* OR workshop	242,788
**#5** (Racial/Ethnic Groups)	minorit* OR “racial and ethnic group*” OR marginalized OR intersectional* OR underrepresented OR disenfranchised OR racialized OR non-white* OR Black* OR “African American*” OR “people of color” OR “colored people” OR negro* OR African OR “Afro-Caribbean*” OR “BIPOC” OR “Black, Indigenous, and People of Color” OR indigenous OR “Native American*” OR “Pacific Islander*” OR Hawaiian* OR “Alaskan* OR Asian* OR oriental* OR Latino* OR Latina* OR Latnix OR “Afro-LatinX” OR “Afro-Latino*” OR “Afro-Latina*” OR “Mexican American*” OR Chicano* OR Chicana* OR Hispanic* OR “Puerto Rican*” OR Cuban* OR “Spanish American*” OR “multi-racial”	250,282
**#6** (People)	scholar OR scholars OR professor* OR faculty OR teacher* OR instructor* OR lecturer* OR adjunct* OR “college student*” OR “community college student*” OR “junior college student*” OR undergraduate* OR “graduate student*” OR “doctoral student*” OR postgraduate OR postdoctoral OR “medical student*” OR “nursing student*” OR attending OR “medical resident*” OR administrat* OR dean* OR provost* OR president* OR “vice-president*” OR “department head*” OR “senior leader*” OR “chief executive officer*” OR staff OR psychologist* OR professional* OR “mental health professional*” OR “mental health personnel” OR physician* OR “medical personnel” OR “health personnel” or “allied health personnel” OR personnel	1,114,567
**#7** (Combined)	#1 AND #2 AND #3 AND #4 AND #5 AND #6	347
Removing dissertations		240

### Selection of sources of evidence

Articles were screened based on the eligibility criteria outlined above. Articles were commonly excluded due to a wrong study design, no evaluation, no strategy/intervention, and no outcomes reported.

### Data charting process

An adapted Covidence Data Extraction template was utilized in this scoping review. One reviewer was responsible for extracting data from eligible studies, another was responsible for verifying the extracted data, and a third was responsible for resolving discrepancies.

### Data items

Elements extracted from each study included the following: title, lead author, aim of study, study design, health science disciplines, institution, location of study, study funding source, participants description, total number of participants, strategy or intervention, comparison group, outcomes assessed, quantitative results, qualitative results.

### Synthesis of results

366 articles met all inclusion criteria and were included in the final scoping review [45].

## Results

### Selection of sources of evidence

**Fig. 1:**
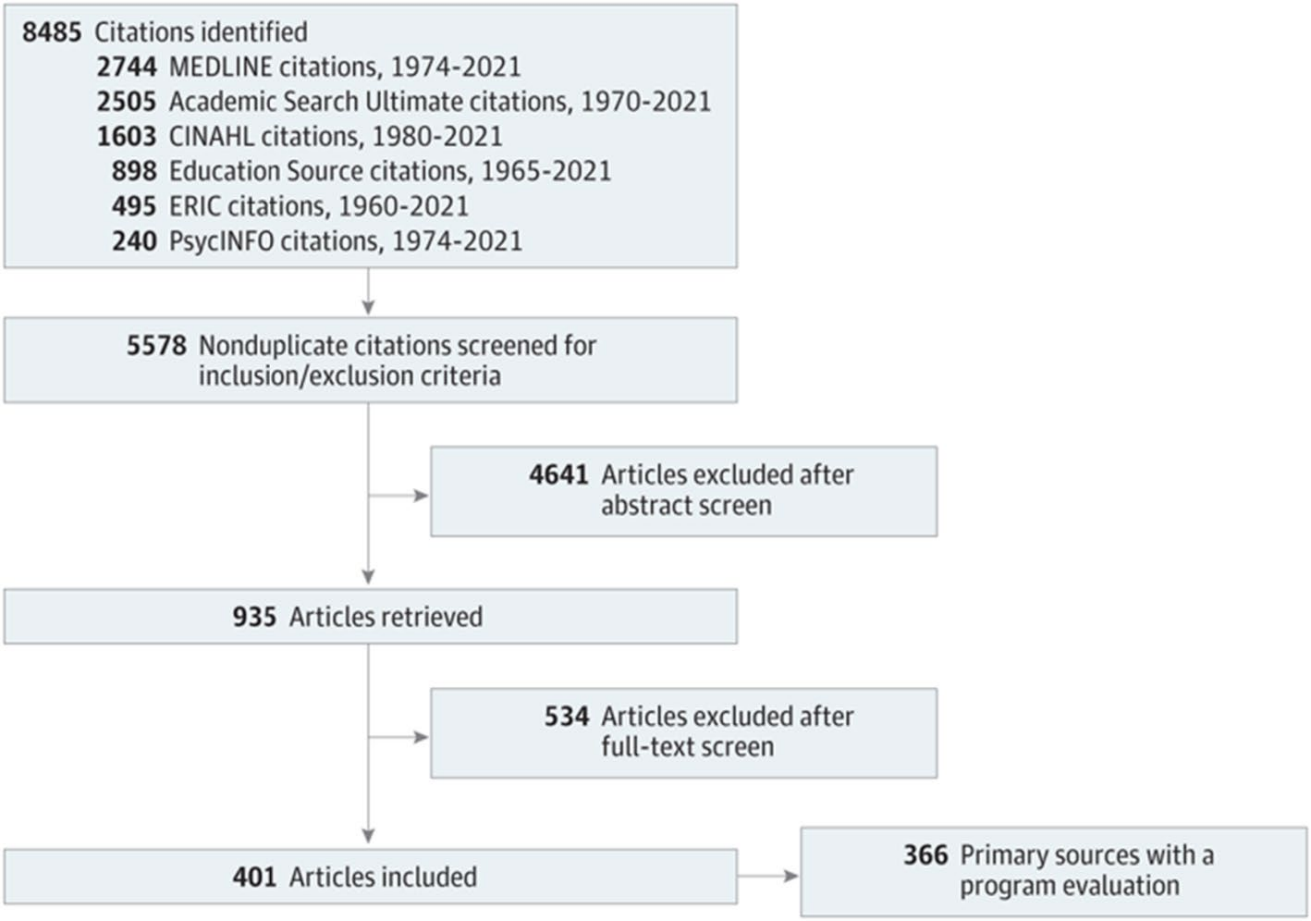
PRISMA Diagram for the Scoping Review. The full texts of 2 article were not able to retrieved after abstracts were seen. Permission to re-use this was obtained from original authors [52][]

A total of 8,485 articles were identified from the initial database search. 5,578 non-duplicates were screened based on inclusion/exclusion criteria. After abstract and full text screening, 401 articles remained. Of the 401 articles, 366 were primary sources with a program evaluation (Fig. 1).

Out of the 366 articles included in the search, six were identified as relevant to institutional partnerships. The six articles identified for the present study were reviewed by the research team to extract specific and overlapping steps for developing institutional partnerships. The articles highlighted three key processes by which institutional partnerships support the recruitment and retention of diverse scholars: 1) HWCUs establishing partnerships with minority serving institutions [46, 47, 55], 2) institutional partnerships creating career advancement opportunities [56, 57], and 3) multi-institutional partnerships as a form of resource sharing [58].

### Institutional partnerships between HWCUs and MSIs

Institutional partnerships between HWCUs and minority serving institutions (MSIs), such as HBCUs and Hispanic Serving Institutions (HSIs), were effective in diversifying the field of health sciences. Partnerships between HWCUs and MSIs resulted in more diverse scholars being accepted into medical school [47], entering specialized medical fields that face a dearth of diverse scholars, such as emergency medicine [46], and becoming leaders in cancer-related health disparities research [55]. Although the elements of each program varied, there were several overlapping commonalities (see Table 2) that may be used to inform future institutional partnerships.

**Table 2:** Steps to forming institutional partnerships for career advancement

	Foote, J. (2006). Health Careers Institute: A Model of Workforce Development. Community College Journal of Research and Practice, 30(2), 129–130. https://doi.org/10.1080/10668920500432969	Roseby, R., Adams, K., Leech, M., Taylor, K., & Campbell, D. (2019). Not just a policy; this is for real. An affirmative action policy to encourage Aboriginal and Torres Strait Islander peoples to seek employment in the health workforce. Intern Med J, 49(7), 908–910. https
Identification of a market-based issue	Identified the need to address issues related to “jobs, housing, and education” for individuals from a low socioeconomic background	Aboriginal people face health related disparities, yet the lack of culturally competent health care providers or those who identify as Aboriginal serve as a major barrier to accessing care
Identification of a goal	Establish a market-based program that addresses issues related to jobs, housing, and education for individuals from a low socioeconomic background	Increase the number of Aboriginal health care workers through an affirmative action policy
Identification of partners	Minneapolis Community and Technical College (MCTC) and various hospitals including Abbott Northeastern Hospital, Children’s Hospitals & Clinics, and Hennepin County Medical Center formed the Health Careers Institute (HCI)	Monash Health, an academic health center, was selected due to 1) the percentage of Aboriginal individuals in the surrounding area and 2) the dearth of Aboriginal employees working at Monash Health. Monash University was selected as the institutional partner. The partnership enabled Aboriginal students at Monash University to have greater job opportunities at Monash Health post-graduation
Definition of Roles	MCTC was tasked with enrolling 1,000 students who want to pursue jobs in the health care field. The partnering hospital institutions were tasked with offering guaranteed jobs to those who graduated	Not clearly defined
Tracking Outcomes	The HCI tracked number of enrolled students, neighborhood of enrolled students, racial and ethnic identification, and wage increases	Monash Health tracked the number of aboriginal employees who had been recruited

Partnerships typically began through the identification of an ideal partner institute that met the designation of an HSI or HBCU [46, 47, 55]. The Undergraduate Medical Academy (UMA) was a partnership established in conjunction between Texas A&M Health Science Center (HWCU) and Prairie View A&M University (HBCU) [47]. Both institutions fell under the larger Texas A&M University system and were within close proximity (i.e., 1-h drive); this was identified as a key component in the success of the partnership.

Engaging additional support staff was identified as an important element in institutional partnerships [46, 47]. In the Morehouse School of Medicine-Emory Emergency Medicine partnership, support staff originally included only the emergency medicine clerkship director but gradually expanded to include other faculty and residents who took on responsibilities such as mentoring [46].

Outlining program goals was a common element across all three institutional partnerships [46, 47, 55]. While some partnerships were established under a single unifying goal, e.g., increasing the number of HBCU students matching into emergency medicine [46], others had multiple specific goals that the program was aiming to achieve [47].

Institutional partnerships included academic, research [47, 55], clinical, social [46, 47, 55], and career [46, 55] supports. Academic support included MCAT preparatory classes, tutoring [47], and courses on health disparities [55]. Research support primarily focused on mentored lab-based research [47, 55]. Clinical opportunities were provided through shadowing experiences [46, 47] and experiential visits to view health inequities in real life settings [55]. Social support was provided through mentorship and role modeling by faculty members [46, 47, 55]. Career guidance included support with writing personal statements [55] and assisting with rank lists for medical students in the matching process [46]. Lastly, all institutional partnerships assessed outcomes associated with the partnerships, including medical school acceptance rates [47], match rates [46], and career progress [55]. 

### Institutional partnerships to create career advancement opportunities

The results of the scoping review also highlighted institutional partnerships as a mechanism for career advancement opportunities for minoritized individuals (see Table 3) [56, 57]. Career advancement partnerships began by identifying a market-based issue and a common and mutually beneficial goal. The Health Careers Institute (HCI) collaborative between the Minneapolis Community and Technical College (MCTC) and various hospitals had the goal of establishing a market-based program that addresses issues related to jobs, housing, and education for low socioeconomic status individuals [56]. Not only did this benefit enrollment rates of the MCTC, but the hospitals benefitted from having a diverse and well-trained workforce. Roseby et al. [57] reported on an affirmative action policy implemented by Monash Health to increase the number of Aboriginal health care providers. Although the primary intervention revolved around an affirmative action policy, Monash Health and Monash University formed a partnership to ensure that Aboriginal students could identify practical post-graduation careers to continue diversifying the field of health care workers. This goal benefitted Monash University students by providing them with possible career advancement opportunities, and Monash Health benefitted by increasing their access to Aboriginal students who could join the workforce. 

**Table 3:** Steps to forming Institutional Partnerships Between Historically white College/Universities and Minority Serving Institutions

	Parrish, A. R., Daniels, D. E., Hester, R. K., & Colenda, C. C. (2008). Addressing medical school diversity through an undergraduate partnership at Texas A&M Health Science Center: A blueprint for success. Academic Medicine, 83(5), 512–515. 10.1097/ACM.0b013e31816be5cf.	Goines, J., Iledare, E., Ander, D., Wallenstein, J., Anachebe, N., Elks, M., Franks, N., White, M., Shayne, P., Henn, M., & Heron, S. L. (2020). A model partnership: Mentoring underrepresented students in medicine (URiM) in emergency medicine. West J Emerg Med, 22(2), 213–217. 10.5811/westjem.2020.9.48923.	Thompson, B., O'Connell, M. A., Peterson, K., Shuster, M., Drennan, M., Loest, H., Holte, S., Simon, J. A., & Unguez, G. A. (2019). Long-term tracking demonstrates effectiveness of a partnership-led training program to advance the careers of biomedical researchers from underrepresented groups. PLoS One, 14(12), e0225894. 10.1371/journal.pone.0225894.
Identifying a partner site	The undergraduate medical academy (UMA) was created via a partnership between the HWCU Texas A&M Health Sciences Center and HBCU Prairie View A&M. Geographical proximity (~ 1 h drive) was critical to establishing this partnership	This partnership was created between HBCU Morehouse School of Medicine and HWCU Emory University School of Medicine	The partnership was created between New Mexico State University (an MSI and HSI) and the Fred Hutchinson Cancer Research Center
Engaging support staff	After its creation, the UMA identified a director who was tasked with liaising between various organizational leaders	Program initially relied on Emory’s department of emergency medicine clerkship director but expanded to include faculty, residents, and a program director	
Community engagement	The UMA created an external advisory board comprised of individuals from academia, government, or health care who shared the common goal of diversifying the health sciences		
Establishing operating structures	The UMA developed an organizational structure that was outlined and incorporated by all major entities		
Outlining program goals	Program leaders were involved in outlining 5 specific goals that the UMA sought to achieve	Increase the number of HBCU students matching into emergency medicine	Increase the number of diverse individuals serving as leaders in cancer and cancer health disparity research
Obtaining funding	The Texas State Legislature funded the UMA; with ongoing efforts to obtain external funding		
Establishing admissions committee	Given that the UMA was a formal program, an admissions committee was created to review applications		
Determining eligibility criteria	Eligibility criteria were based on educational classification (‘premed’) and grade point average (GPA)		
Determining application criteria	Application materials included a personal statement and letters of recommendation		
Determining admission criteria	Admission decisions were based on minimum GPA requirements, personal statement, letters of recommendation, and interview performance. A class size of 20–25 was deemed the most optimal		
Academic elements of the partnership	Academic elements included academic counseling, tutoring, and MCAT preparatory classes		A biostatistics workshop for graduate students and health disparities courses were offered through Fred Hutch. A post-baccalaureate program was also hosted
Career advancement elements of the partnership		Morehouse students received career guidance, help with rankings, and support for match applications	Support writing personal statements
Clinical elements of the partnership	Seminar series in which Texas A&M College of Medicine faculty and students presented on various topics and clinical specialties. UMA students were provided opportunities to shadow clinicians	Morehouse students were provided with shadowing opportunities and given priority for Emory’s emergency medicine rotation	Border Experiences Program which involved an experiential visit to the New Mexico-Mexico border to view health inequities in real life setting
Research elements of the partnership	UMA students participated in a six-week biomedical research experience working with faculty at Texas A&M College of Medicine		Summer internship for NMSU undergraduate and graduate students to gain exposure conducting research. Undergraduate students worked in a faculty member’s lab and produced a research poster at the end of the internship. Encouraging undergraduate and graduate students to work in cancer-focused research labs during the school year. Opportunities to participate in the peer review process
Social support elements of the partnership	Students from the Texas A&M College of Medicine visited with UMA students which provided opportunities for medical students to teach, mentor, and serve as role models. This was viewed as one of the most important elements of the partnership	Students from Morehouse School of Medicine were able to access faculty members within the Emory emergency medicine department. Faculty mentors Underrepresented in Medicine (UriM) at Emory served as role models for Morehouse students who were UriM	Social relationships were developed with students participating in the partnership as well as interns working at Fred Hutch. Students also received mentorship
Measurable outcomes	The UMA partnership assessed retention and medical school acceptance rates	Match rate into emergency medicine	Racial and ethnic identification, degree completion, topics of dissertations, occupation, cancer biology and cancer disparity knowledge. Additionally, the program collected qualitative responses

The identification of partner institutions that could fulfill the goals outlined was an important process in both studies [56, 57]. Given the goal of the HCI collaborative to establish a market-based program that allowed people to obtain future employment opportunities, a partnership between a technical college and hospital was ideal [56]. Given that the overarching goal of the Monash Health–Monash University partnership was to increase the number of Aboriginal health care workers, a partnership between a health services provider and health education institution worked in tandem to accomplish this goal. Monash Health was selected due to the percentage of Aboriginal individuals in the surrounding area and the dearth of Aboriginal employees working at Monash Health. Monash University was selected due to their ability to bridge academic-career pathways and ensure that Aboriginal students had greater job opportunities at Monash Health post-graduation [57].

Identifying roles for the institutions was an important element in these partnerships. For example, in the HCI partnership between MCTC and various hospitals, the specific role of MCTC was to enroll 1,000 students, while the hospitals’ role was to provide jobs to MCTC graduates [56].

Tracking outcomes and metrics was another important aspect of these partnerships. Outcomes such as enrollment rate, neighborhood of enrolled students, racial/ethnic identifiers, wages, and employment outcomes were documented [56, 57]. In the HCI, the authors reported that 1,000 students had been enrolled, 80% of whom identified as people of color. The HCI also reported a 27% wage increase among those who participated compared to previous jobs [56]. Monash Health reported that the number of Aboriginal employees recruited increased from 24 to 57 in a 12-month period [57].

### Institutional partnerships as a form of resource sharing

The PROMISE alliance was a multi-institutional partnership between the University of Maryland, Baltimore County (UMBC), the University of Maryland, College Park (UMCP), and the University of Maryland, Baltimore (UMB) in response to the relatively low enrollment and graduation rates of graduate level URMs at UMBC [58]. The goal of the program was to increase the recruitment of undergraduate URM scholars into graduate programs, retain graduate students, and increase graduation rates of URM graduate students. The PROMISE alliance included mentorship, community-building, peer mentoring, training workshops, and recruitment activities focused on minoritized populations.

The partnership began with identifying gaps in URM scholars' recruitment and graduation rates. Between 1996–1997 and 2001–2002 academic years, UBMC had an annual average of 35 URMs enrolled in graduate programs and 8 earning MS and PhD degrees, compared to 535 and 171 students of other races (including international students), respectively. Institutional qualities played a role, such that the partnership was established between three institutions within the same university system and were in physical proximity to one another, which was identified as an essential factor in creating successful partnerships and allowing for cross-institutional work.

The partnership was also shaped by a shared goal across the three partnering institutions: “to recruit, retain, graduate, and transition students to STEM careers, with special emphasis on the professoriate” [58]. One of the first steps in establishing the partnership was establishing a leadership team and roles. In the PROMISE collaborative, the leadership team was comprised of faculty co-PIs, the program director, and PROMISE coordinators. Each of whom served distinct roles within the collaborative: co-PIs were responsible for providing administrative leadership for their campus and implementing and assessing program initiatives, the program director oversaw cross-institutional events, and the PROMISE coordinators worked at their specific institution.

The partnership was also unique in integrating and leveraging the resources of multiple universities to provide activities related to recruitment, retention, graduation, and career transition. During the early stages of the PROMISE collaborative, the “Success 2003” initiative included visiting the various campuses and participating in seminars. Although the initiative ended, recruitment, retention, and graduation efforts still leverage resources from the three campuses. Recruitment efforts included campus visits, seminars, and networking. Retention efforts focused on professional development and community building. Graduation efforts included dissertation support and opportunities to attend conferences. Career preparation efforts included networking and mentorship.

Lastly, the PROMISE collaborative assessed for pre-post changes in targeted outcomes, including applications, enrollment, and graduation. Qualitative results from focus groups highlighted the role of PROMISE in supporting students’ professional development, creating avenues for emotional support with other scholars. Quantitative enrollment and graduation rates also indicated program success. URM scholars' average annual enrollment and graduation rates between 2002–2003 and 2008–2009 were 112 and 27, respectively. Compared to before the implementation of PROMISE, schools in the alliance saw a 45% increase in URM applications, a 44% increase in URM enrollment, and a 45% increase in PhD graduates.

## Discussion

Findings from the existing literature provide practical and best practice guidance for establishing institutional partnerships to increase racial and ethnic diversity in the academic health sciences. Three primary reasons for establishing institutional partnerships emerged. First, partnerships between historically white colleges and universities and minority serving institutions occurred to increase enrollment of URMs [46, 47, 55]. Second, partnerships created career advancement opportunities for minoritized individuals [56, 57]. Third, partnerships were a form of resource sharing across linked institutions [58]. Across the 3 types of institutional partnerships were several commonalities, including identifying a partner site, outlining shared goals, and tracking measurable outcomes [46, 47, 55–58]. When establishing a partnership, physical proximity emerged as an essential consideration [47, 58]. Career advancement elements were incorporated in five partnerships [46, 55–58] and thus appear to be a key component in diversifying the academic health sciences.

Findings support Toretsky and colleagues’ [48] “Framework to Successfully Recruit and Retain URMs in Health Professions.” Not only were institutional partnerships associated with diverse outcomes, but several partnerships involved other components proposed by the framework, including academic, career, and social support. The present study highlights existing literature that maps onto the framework, provides support for the framework, and outlines guidance for future research on institutional partnerships to diversify the academic health sciences.

### Policy implications

The findings from this review are particularly relevant given the Supreme Court rulings and anti-diversity, equity, and inclusion (DEI) legislation that threaten initiatives to diversify academic health sciences across the United States. According to the Chronicle of Higher Education [59], since 2023, 119 anti-DEI legislation have been proposed, with 14 becoming law. This includes restrictions on DEI offices and staff, mandatory DEI training, diversity statements, and identity-based preferences for hiring and admissions.

Within healthcare, recent anti-DEI efforts include challenges to medical board policies on implicit bias training and representation, eliminating the ability for providers to be rewarded for anti-racism initiatives, and proposed legislation to defund medical schools with DEI programs [60]. The threat of lawsuits has also lead to the abandonment of programs that were offering financial incentives or fellowships to racial and ethnic minoritized physicians and students in an effort to diversify the field [60]. The consequences of such efforts are evident, with the Association of American Medical Colleges [61] reporting a decrease in the percentage of enrolled URM students for the 2024–2025 academic year.

Anti-DEI efforts effectively limit the tools that institutions can harness to create inclusive environments and maintain diverse student bodies, emphasizing the urgency to focus on new methods. For example, although the U.S. Supreme Court ruled to limit race-conscious admissions significantly, race-consciousness may be utilized in other supports for minoritized students, such as hosting admissions events, creating financial, academic, and social support programs, as well as peer networking and career development opportunities [62]. Partnerships with institutions, community organizations, and healthcare providers can play a significant role in amplifying the reach of efforts to diversify the academic health sciences through sharing best practices, resources, and supports to form sustainable efforts that are less vulnerable to legal and political changes [48, 62].

### Limitations and future directions

The number of articles included in this review is low. Of the 366 articles identified as part of the larger scoping review [45], only 6 were relevant to institutional partnerships and thus included in the present paper. In addition to increasing the number of articles examining institutional partnerships, this literature would benefit from a more empirical and comprehensive model of institutional partnership creation and sustainability. This area was rarely discussed in the published papers. There was also a dearth of literature examining the association between compositional diversity and learning outcomes in fields outside of medicine. Future research should examine more nuanced associations related to nursing and dentistry. The present review relied on a secondary approach to data analysis. It is possible that some articles utilizing institutional partnerships were not captured in our review, given that the original scoping review aimed to examine evidence-based approaches to increasing diversity, equity, and inclusion broadly [45]. Furthermore, the scoping review was conducted in 2021, which means that our review may not capture the full extent of the legislative and political shifts that have impacted DEI efforts in subsequent years. Specific steps towards establishing institutional partnerships highlighted in Table 2 and Table 3 may need to be modified to fit within the existing political climate. Future research is needed to understand how institutional partnerships are impacted by new DEI regulations imposed at the university, state, and federal levels. The methodological rigor of the included studies could be improved upon, as there were no randomized controlled trials. Future research should also employ pre- and post-test assessments of their outcome measures of interest. Although institutional partnerships may be an effective tool to diversify the academic health sciences, establishing working relationships between HWCUs and MSIs such as HBCUs may also pose its own set of challenges and occurs within a historical context of long-standing colonization and imperialism. Specifically, while there are benefits of such partnerships, there are also numerous difficulties. These include the devaluing of community-engaged research, a lack of transparency from HWCUs on what is expected from MSIs and how ideas will be used, pressures on MSIs to assimilate to HWCU culture, co-opting ideas originating from HBCUs leading to intellectual theft and appropriation, lack of sustainability [63], and the delegitimization and social exclusion of HBCUs by HWCUs [64]. Lastly, the order in which elements of the partnerships occurred was not always clear and had to be inferred by the team. Future research should include a more robust documentation of the steps taken and the order in which they occurred when forming institutional partnerships; methodological transparency will enable other researchers to replicate and test these strategies in different settings.

## Conclusion

Uncertainty around best practices for institutional partnerships have remained at the core of the existing literature. The present study gathers the best practices identifiable in the current literature, consistently highlighting the collective benefits of diversifying the academic health sciences [15, 22] and was supported by this review. The best practices identified provide promising mechanisms to diversify the academic health sciences and enhance the experiences of health professionals and the patients they serve.

## Data Availability

The datasets used and/or analyzed during the current study are available from the corresponding author upon reasonable request.

## Footnotes

1 “Also known as people of the global majority (PGM), a collective term that encourages those of African, Asian, Latin American, and Arab descent to recognize that together they comprise the vast majority (around 80%) of people in the world. Understanding the truth that Whiteness is not the global norm has the power to disrupt and reframe conversations on race” [7].
